# Regeneration of the Retina Using Pluripotent Stem Cells: A Comprehensive Review

**DOI:** 10.7759/cureus.53479

**Published:** 2024-02-02

**Authors:** Yash V Lath, Archana R Thool, Indrayani Jadhav

**Affiliations:** 1 Medicine and Surgery, Jawaharlal Nehru Medical College, Datta Meghe Institute of Medical Sciences, Wardha, IND; 2 Ophthalmology, Jawaharlal Nehru Medical College, Datta Meghe Institute of Medical Sciences, Wardha, IND

**Keywords:** efficacy, safety, bioengineering, transplantation techniques, differentiation strategies, pluripotent stem cells, retinal regeneration

## Abstract

Retinitis pigmentosa and age-related macular degeneration are the most frequent causes of irreversible visual impairment in the world. Existing therapeutic methods could be more effective, underscoring the necessity of new treatments. Reconstructing the retinal photoreceptors through the transplantation of human pluripotent stem cells, representing an attractive approach for restoring vision, has gained momentum. This paper gives an exhaustive account of what has been known in this field, the discoveries made, and the recent progress. This review paper outlines the retina's organisation, cell types, the pathophysiology of retinal injury/degeneration, and the reasoning behind using pluripotent stem cells in retinal regeneration. This article investigates differentiation strategies, molecular components that dictate cell type specification, and the recreation of retinal development in vitro, genetically engineering and manipulating epigenetic marks using various techniques for driving specific cell fates and improving therapy efficacy.

Subretinal injection methods, cell encapsulation techniques, scaffold-based approaches, cell sheet transplantation, and their impact on integrating implanted cells into a functional retina are thoroughly reviewed. Using bioengineering approaches, biomaterials and growth factors form a favourable micro-ambience for grafted cells. Issues around safety and efficacy (tumorigenicity, immunological rejection, and long-term integration/functionality) are explored. Moreover, the paper emphasises the significance of rigorous characterisation, immunomodulatory strategies, and clinical and pre-clinical studies to ensure the safety and effectiveness of retinal regeneration therapy. Future perspectives and challenges are presented, looking at fine-tuning differentiation strategies, improving functional integration and regulatory aspects, and using co-therapy and supportive treatments.

## Introduction and background

The eye's retina is an intricate and fragile part of our body that helps us see by converting light energy into electric energy impulses. However, numerous eye diseases, like age-related macular degeneration, retinitis pigmentosa (RP), and diabetic retinopathy, may cause permanent harm, resulting in vision loss that can be unrecoverable. Traditional approaches like medicines and gene therapy (Luxturna, an FDA-approved gene therapy modality for RPE 65-induced RP) for treating RP do contribute much to the functionality of the patients; however, newer modalities of treatment are needed to regain vision in those cases where even the traditional efficacious medicine fails to treat the patient. One option for further study appears to use pluripotent stem cells to regenerate the retina [1].

Retinal regeneration is the recovery or renewal of damaged or lost retinal cells to restore vision. That is why pluripotent stem cells can take on virtually any form of cell within the human body, such as retinal cells. They represent a large field in regenerative medicine since embryonic stem cells (ESCs) and induced pluripotent stem cells (iPSCs) are some sources that can generate them. These cells show potential for repairing or replacing the damaged retinal tissue, which could improve vision in people with retinal degenerative diseases [2].

Retinal regeneration is important because many common eye disorders severely affect people's lives. The WHO estimates that around 285 million people around the globe experience visual impairment, and almost one-third of these cases are linked to retinal diseases. This can significantly impair individuals' capacity for performing everyday tasks and cause psychological and societal repercussions. The growing prevalence of eye diseases among the geriatric population demands new therapeutic strategies which could recover visual functionality [3].

With pluripotent stem cells' capability to differentiate into various cell types in the retina, such as photoreceptors, retinal pigmented epithelium (RPE), and retinal ganglion cells (RGCs), the hope for the future is to produce an infinite number of these cells for use in tissue growth and organ donation initiatives. In addition, pluripotent stem cells surpass the deficient regrowth capability of adult rodents' retinas and do not rebuild those cells to enough extent [2].

This article aims to clarify the current understanding of retinal regeneration utilising pluripotent stem cells. It will explore pluripotent stem cells' characteristics, ways for generating retinal cells from them, transplantation techniques, safety considerations, potential drawbacks, and the field's bright future. Pluripotent stem cells have the ability to shed light on retinal regeneration, which will aid in the development of more potent treatments to restore vision for all patients with retinal degenerative illnesses.

## Review

Structure and cell types of the retina

The retina is one of the most important parts of the eye, a thin, delicate tissue that forms layers in the back of our eyes. Light is converted into electrical signals sent to the brain to be processed as vision. The retina has multiple layers, and the cells in each layer have a specific function for vision processing.

The retinal pigment epithelium (RPE), which makes up the outermost layer of the retina, is a single layer of pigmented cells that supports and feeds the photoreceptor cells that lie on top of it while also eliminating waste. The two main types of sensory cells in the retina are called photoreceptors: rods and cones. Cones are in charge of photopic vision, which is typical, bright-light, full-colour vision; rods are in charge of scotopic vision, which is low-light or dim-light vision in black and white. The bipolar cell layer receives its stimulation from the photoreceptor layer, i.e., the rods and cones, and sends those electrical impulses to the ganglion cell layer.

There are many cell types found in the inner nuclear layer, including amacrine, horizontal, and bipolar cells. Bipolar cells in the next layer communicate with the ganglion cells by receiving information from photoreceptors. These include amacrine and horizontal cells, which help in spatial encoding by processing these signals through lateral inhibition.

A layer of ganglion cells is made up of several hundred ganglion cells, which are the retina's ultimate output neurons. These signals are sent by bipolar cells to ganglion cells, which then send their axons via the optic nerve and out of the eye. The optic nerve then sends this visual data to the brain for additional processing [4].

Mechanisms of retinal damage and degeneration

Damage and degeneration can affect the retina in numerous ways, causing loss of function, a decline in vision, or even total blindness. Retinal disease can be due to many reasons, like genetic mutation, environment-based issues, oxidative stress, inflammation, and vascular aberrations. The specific pathways of retinal damage are different for different diseases/conditions. Nevertheless, some specific paths and processes have repeatedly been referred to as the commonest causative mechanisms.

Age-related macular degeneration (AMD) and RP are two retinal degenerative disorders that are particularly prone to damaging photoreceptor cells, particularly the rods and cones. The gradual loss of photoreceptors can be attributed to genetic abnormalities that disrupt key genes, such as those involved in phototransduction, photoreceptor structure, and cellular maintenance pathways. Photoreceptor degeneration may also result from malfunctioning of the RPE, which supplies nourishment and support to the photoreceptors [5].

Some abnormal changes are observed in our blood vessel structure, including the retinal vascular network, which has also contributed to causing damage to our retina. These conditions, diabetic retinopathy and retinal vein occlusion, involve impaired blood flow, fluid leakage, and the growth of irregular blood vessels within the retina. Such vascular changes may cause retinal hypoxia, inflammation, and oxidative injury, eventually resulting in cellular malfunction and cell death [6].

The rationale for regenerative approaches

The finite regenerative capacities of the mammalian retina highlight the necessity of regenerative strategies in tackling retinal degenerative conditions. Conventional treatments like medications and surgical procedures aim to manage the symptoms or slow down the disease progression instead of reversing visual loss. In contrast, regenerative tactics focus on either regrowing lost retinal cells or supplying better-formed replacement cells, with an eye towards improving the sharpness of vision and improving the quality of life.

Pluripotent stem cells provide an exciting path towards retinal regeneration because of their exceptional capacity to develop into many kinds of retinal cells. These cells may be directed to develop into RPE cells, functioning photoreceptors, and other kinds of retinal cells in culture. These cells may integrate into the tissue after being transplanted back into the sick retina, replacing any lost or damaged cells and restoring eyesight. Furthermore, personalised treatments through the use of patient-specific iPSCs are made possible by regenerative procedures [7].

Stem cells can be created from a patient's cells and converted to an earlier form of cell called pluripotent, which could be further converted to the desired type of cells called retinal cells. This technique also lowers the chances of immune rejection, customises a therapy plan, and has potential benefits in future medical treatments or disease areas.

Targeted regenerative solutions can be built by understanding how the eye loses function due to the various damaging factors in retinal injury and degeneration. The motivation behind using regenerative medicine for retinal disorders comes from the opportunity to replace damaged retina cells, which can bring back the lost vision by maintaining the existing retinal circuitry or reconnecting the residual one, which might lead to better treatment outcomes in contrast to traditional approaches, which might fall short on improving or restoring the visual acuity of a diseased individual.

Pluripotent stem cells

An advanced class of stem cells known as pluripotent stem cells has the ability to differentiate into any other type of cell in the body. Reprogrammable stem cells have great promise for in vitro production of many specialised cells, including retinal lineage cells, which may find application in regenerative medicine. ESCs and iPSCs are the two primary categories into which pluripotent stem cells may be roughly divided. They have distinct qualities that are clinically relevant for research and therapy [8].

ESCs

The interior cell mass of the blastocyst, an immature pre-embryo stage, is where ESCs begin. Because they still have the ability to differentiate into cells from the ectoderm, mesoderm, and endoderm, these cells are regarded as pluripotent. ESCs are distinct in a number of ways.

Self-renewal: ESCs are capable of endless division and proliferation in culture while still pluripotent. This characteristic guarantees a constant and renewable supply of cells for analysis and possible therapeutic uses.

Pluripotency: ESCs are useful for researching early human development and producing certain cell types for transplantation or disease models since they can differentiate into cells of all three germ layers [9].

iPSCs

iPSCs are derived from a somatic cell (usually skin) that's been genetically reprogrammed with the help of viral vectors or some other delivery method and injected with specialised transcription factors. The most broadly utilised restoring elements incorporate OCT4, SOX2, KLF4, and c-MYC. This is done by changes in the epigenetic landscape and gene expression profiling of the somatic cells that result in turning the somatic cells into more or less pluripotent states looking like ESCs. That groundbreaking achievement, realised in 2006, bypasses one level of discomfort with embryo-derived ESCs. iPSCs possess the following characteristics.

Reprogramming: By adding certain transcription factors or reprogramming factors that cause a pluripotent state, somatic cells can be transformed into iPSCs. These elements can revert the cellular identity, wiping out the somatic cell traits and activating pluripotency-related genes.

Pluripotency: Because iPSCs can differentiate into cells from all three germ layers, just as ESCs, they are an important resource for disease models, drug research, and regenerative medicine [7].

Characteristics of Pluripotent Stem Cells

Pluripotent stem cells, whether ESCs or iPSCs, share common characteristics that distinguish them from other stem cell types.

Pluripotency: Pluripotent stem cells can form cells belonging to all three embryonic germ layers, ectoderm, mesoderm, and endoderm.

Self-renewal: Pluripotent stem cells can replicate indefinitely while maintaining their pluripotency, so there is always an endless supply of undifferentiated cells.

Marker expression: These stem cell lines are characterised by expressing specific cell surface markers that identify them as pluripotent, including SSEA-4, OCT4, and several others. We can use these markers to locate, separate, and purify pluripotent stem cells.

Epigenetic state: Such cells have a relaxed and heterogeneous chromatin structure, repositionable for gene expression of many types. This flexibility allows the cells to form various cellular types [10].

Ethical Considerations

The production and utilisation of pluripotent stem cells, especially in ESC, presents an ethical issue due to their relation with embryos. Following is a summary of the ethical issues regarding pluripotent stem cells:

Embryo destruction: The destruction of human embryos in order to create ESCs is an issue which raises questions of morality since ESCs may carry the potential to create life.

Informed consent: To use a donor's pluripotent stem cells, it is obligatory to get prior approval of this person through a process called informed consent with regard to making sure that all parties involved understand the objectives, risks, and benefits associated with the trial.

Alternative approaches: With the advent of iPSCs, there is now a morally acceptable alternative to ESCs derived from destroying human embryos. With iPSCs, somatic cells may be directly reprogrammed to become pluripotent cells without the need for embryos.

Regulatory frameworks: Many countries have enacted laws and guidelines in response to moral questions about pluripotent stem cell research. Employing these frameworks will guarantee ethical and responsible behaviour, including obtaining consent from donors, supervision over the investigation of stem cells, and open access to stem cell technology [11].

Pluripotent stem cells, such as ESCs and iPSCs, offer enormous promise for drug development, disease models, and regenerative medicine. ESCs symbolise the pluripotency derived from early-stage embryos, even if iPSCs are an ethically acceptable method of creating pluripotent cells from adult tissues. Before we use pluripotent stem cells effectively and responsibly in research for scientific understanding and therapeutics, we must understand their properties and address the related ethical issues [12].

Production of retinal cells using pluripotent stem cells

Producing retinal cells from pluripotent stem cells, such as ESCs and iPSCs, for regenerative medicine and studying retinal development and diseases provides opportunities and challenges that warrant further exploration.

Differentiation Approaches

Different methods for guiding pluripotent stem cells towards retinal cell fates were developed. They attempt to recreate the normal in-vivo retina development during gestation. The following are the important methods.

Differed differentiation: To accomplish directed differentiation, appropriate growth stimulants, signalling molecules, and culture conditions reflecting the retinal development environment should be implemented in sequence. For example, the early administration of activin A and bone morphogenetic protein 4 (BMP4) induces the formation of structures that resemble the eye field. Retinoic acid (RA) and fibroblast growth factor (FGF) are applied in two stages, including the development of retinal progenitor cells (RPCs), which express such markers as PAX 6, LHX 2, and SIX 3 [9]. Table 1 depicts retinal cell differentiation markers [13].

**Table 1:** Retinal cell differentiation markers This table gives an outline and mentions the biomarkers for retinal cell differentiation. Different biomarkers are utilized for different stages of development. RPE: retinal pigment epithelium; RHO: rhodopsin Table Credit: Weed et al., 2017 [13]; Licensed under CC-BY

Stage of development	Marker
Anterior neuroepithelium	PAX6, OTX2
Eye field	RAX, PAX6, LHX2, SIX3, SIX6
Optic vesicle	PAX6, MITF, VSX2
RPE progenitor	PAX6, MITF
Neuroretinal progenitor	PAX6, VSX2
Photoreceptor progenitor	CRX, RCVRN
Mature RPE	MITF, BEST1, ZO-1, CRALBP, PEDF, PMEL17
Mature cone photoreceptor	Cone arrestin, red-opsin, blue-green opsin
Mature rod photoreceptor	RHO, NRL
Retinal ganglion cell	BRN3, HuD
Müller glia	GLUL

Co-culture systems: Integration of pluripotent stem cells to retinal-specific cell types, such as RPE or retina progenitor cells, provide important signalling signals and physical interaction to such cells. This strategy promotes the expression of retinal biomarkers such as OTX2, CRX and NRL, leading to better retina development [14].

**Figure 1:**
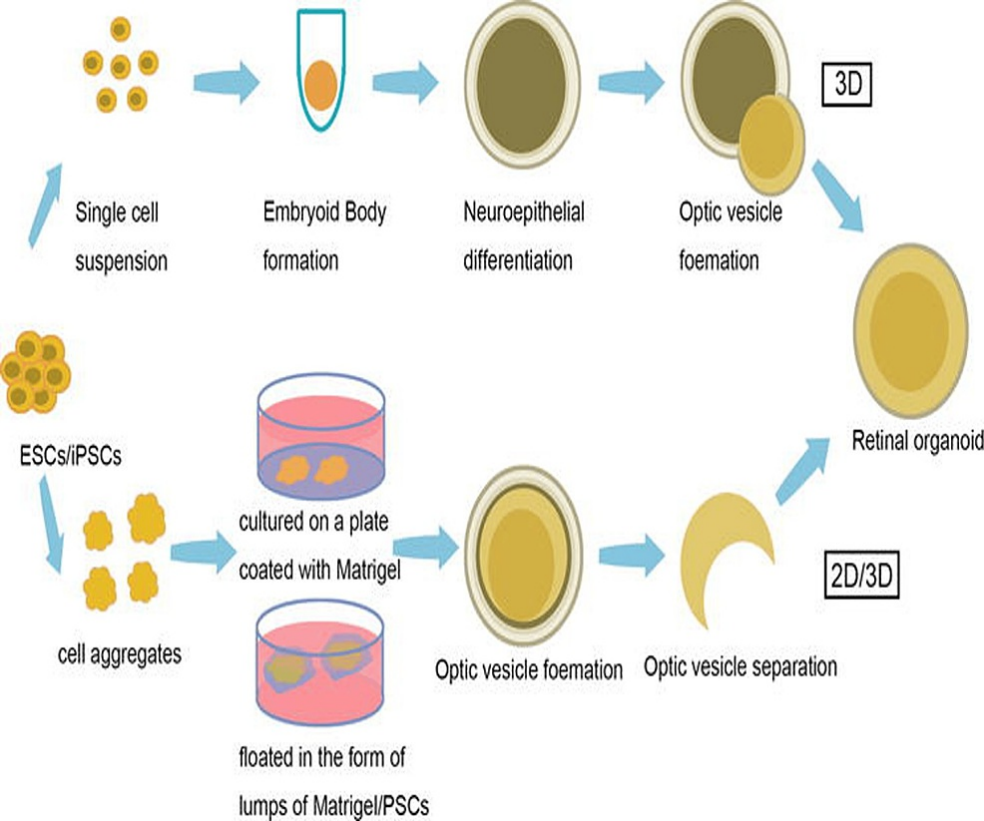
Main techniques of retinal organoid development Image Credit: Yuan et al., 2022 [15]; Licensed under CC-BY

Three-dimensional (3D) organoid culture: Creating 3D retinal organoids enables recapitulating retinal development in vitro. These organoids exhibit a cellular complexity and layered architecture similar to the developing rat retina. Three-dimensional retinal organoids express markers indicative of retinal cell types, including photoreceptor markers (e.g., CRX, RCVRN), bipolar cell markers (e.g., PKCα, CALB2), and Müller glial cell markers (e.g., GLUL, GFAP) [15]. Figure 1 depicts the main techniques of retinal organoid development [15].

Factors affecting retinal cell differentiation

Many variables impact the ability of pluripotent stem cells to differentiate successfully into retinal cells.

Temporal Regulation

These cells must be exposed early to specific signalling molecules and growth factors that direct them towards a retinal fate before being pluripotent. For example, Wnt signalling is responsible for determining the fate of retinal cells at specific stages. Early suppression of Wnt signalling induces the development of optic cup-like structures, and later activation enhances photoreceptor determination [16].

Signalling Pathways

Several signalling pathways play an important role in retinal development, with some being modulable to regulate pluripotent stem cell differentiation. An example of this is how Notch signalling helps maintain RPCs and keeps them from differentiating prematurely into neurons. When the ligand DLL4 activates Notch signalling, markers such as HES1 and SOX2 are expressed [17].

Transcription Factors 

In pluripotent stem cells, transcription factors can upregulate the expression of retinal genes and aid in the formation of the retina. In fact, PAX6 is one instance of a master regulatory gene that regulates retinal development; when it is overexpressed, it can cause retinal cells to differentiate. Different transcription factors that contribute to the development of particular kinds of retinal cells include RX, NRL, and CRX [18].

Recapitulation of retinal development in vitro

Pluripotent stem cells have been tried for in-vitro retinal development, yet there is complexity involved in this process. The stem cell lineages of pluripotent stem cells can sequentially develop through various stages of retinal development, mimicking the natural in-vivo process merely by altering the cultured conditions and exposing them to the specific stimulus for each stage. These stages include the formation of optic vesicles, the generation of retinal progenitor cells, the emergence of characteristic retinal cell types, and finally, the establishment of multi-layered retinal structures.

Retinal Development Recapitulated in a Laboratory Setting as a Model for the Study of Key Developmental Processes, including Cell Fate Determination, Cell-Cell Interactions, and Morphogenesis

The activation of transcription factors such as PAX6, SIX3, and LHX2 is necessary to identify eye progenitors expressing marker genes, including VSX2, SOX2, and PROX1, in developing optic cups [19].

Genetic manipulation and epigenetic regulation

Creating Retinal Cells From Pluripotent Stem Cells

The role of genetic engineering and epigenetic control: These strategies facilitate the exploration of gene function in retinal development and disease and enhance efficiency and specificity in retinal cell differentiation.

Genetic manipulation: Genetic manipulation methods such as gene overexpression, knockdown, or knockout can change the expression of specific genes controlling retinal development. To illustrate, the overexpression of transcription factors such as PAX6 RX or CRX is known to boost the production or generation of certain retinal cells or cell types. A potential approach could be targeting genes such as Notch1 or Hes1 using knockdown techniques which interfere with the signalling pathways, effectively changing the fate of retina cells [20].

Epigenetic regulation: Epigenetic changes, including DNA methylation, histone modifications, and non-coding RNA expression, act upon gene expression patterns during development. To manipulate these epigenetic markers to control how pluripotent stem cells differentiate into retinal lineages. For example, one may use DNA methyltransferase inhibitors to alter DNA methylation patterns and promote histone acetylation for retinal cell differentiation; otherwise, one may use histone deacetylase inhibitors to achieve the same objective [21].

Pluripotent stem cell-generated retinal cells have significantly improved our understanding of retinal development, and they also hold promise for regenerative medicine and disease models. By utilising various approaches such as differentiation strategies taking into account factors affecting retinal cell differentiation, simulating retinal development in vitro, and employing genetic manipulation and epigenetic regulation, pluripotent stem cells can be directed towards differentiating into consequently specific cell types needed for retinal regeneration; our deep scientific understanding allows us to comprehend the complexities of the retinal development, delve deep into the sources of various diseases, and develop novel treatments to solve the prevailing issues in the retina field.

Pluripotent stem cell transplantation methods

Cell sheet transplantation, encapsulation and scaffold-based approaches, subretinal injections, and bioengineering techniques are among the transplantation procedures available. The irreversible loss of photoreceptor cells brought on by retinal degenerative diseases such as AMD and RP results in vision loss. Regenerative medicine uses pluripotent stem cells, such as iPSCs and ESCs, to replace damaged or killed retinal cells in an effort to restore vision [22].

Subretinal Injections

The transplantation of retinal cells into the subretinal space involves injecting them directly into the eye cavity. This way, cells can be delivered straight to the target site for better uptake by the host retina. Surgical supervision is required for subretinal injections through a thin-gauge or microcannula needle. In subretinal transplantation, four key elements, namely cell survival, proper cell density, and avoiding retinal detachment or injury concerning surgery, can be identified as essential for a successful outcome. Research has indicated that subretinal injection of retinal lines generated from pluripotent stem cells, for instance, photoreceptor precursor cells, could retrieve the sight in animal models of retinal deterioration. The engrafted cells mature into the host retina, acquire a functional retinal cell phenotype, and establish synaptic connectivity with the remaining retinal machinery [23].

Encapsulation and Scaffold-Based Approaches

Retinal cells produced from pluripotent stem cells are contained using biocompatible materials or scaffolds in encapsulation and scaffold-based techniques. These tactics guide the transplanted cells' maturation and integration into the retina while maintaining their physical connection and protecting them from the host immune response.

To protect transplanted cells, hydrogel or semi-permeable membrane encapsulation techniques are used. These substances prevent the entry of immune cells into the tumours while allowing for proper blood circulation. Encapsulation enhances cell survival and long-term functional integration of transplanted cells [24].

In scaffold-based methods, three-dimensional (3D) structures that contain retinal cells derived from pluripotent cells and biocompatible materials support these cells and organise them in a manner that resembles a natural retinal environment.

Scaffolds made from biodegradable materials like polymers or decellularised extracellular matrix can provide structural guidance and direction for cell growth and tissue regeneration. The 3D geometry of scaffolds promotes the development of organoid-like retinal structures and supports transplanted cells' maturity and integration [25].

Cell Sheet Transplantation

Transplanting whole sheets of retinal cells produced from pluripotent stem cells is known as a cell sheet transplant. The cells are grown until they form a complete sheet, which may then be collected. Cell integration, existence, and functionality are made possible by cell sheets, which guarantee the continuity of the relationship between the cells and the matrix. A great deal of transplantation is made possible by cell sheet transplantation, which is simple to handle and maintains cell-cell connection. The intact cell sheets can be placed on a carrier membrane for a subsequent transplant or directly onto the host retina. Research on animals has shown that transplanting retinal cell sheets made from pluripotent stem cells helps animal models regain their vision [26].

Bioengineering Strategies

To enhance the long-term survival, maturation and engraftment of pluripotent stem cell-derived retinal cells, bioengineering technologies incorporate these cells on biomaterials, growth factors, or gene therapy approaches. The goal is to create the ideal environment for the reintegration of retinal cells in a functional context [27].

Biomaterial-based techniques involve incorporating growth factors or components of extracellular matrix into scaffolds or encapsulation systems. Growth factors promote these cells' survival, division, and differentiation, whereas non-biological but equally important features include extracellular matrix (ECM), which provides structural integrity and orientation [28].

Using gene therapy techniques, one can modify host variables to render hospitable surroundings and enhance the functional features of transplanted cells. Viral-mediated gene delivery or genome editing can add certain genes for improved cell survival, integration and functioning [29].

Treating retinal degenerative illnesses and restoring visual capabilities are potential future uses for totipotent stem cell transplantation in retinal regeneration therapy. Subretinal injection-based retinal cell transplantation in vivo is not the same as scaffold-based methods or encapsulation techniques that are intended to establish safe environments and signals for cell fusion. Cell sheet transplantation can enable large-scale organ donorship by retaining cell-cell connections. Bioengineering techniques use biomaterials, growth factors, or gene therapy to increase cell survival, maturation, and integration [26].

Such evidence-based transplantation techniques are likely to lead to the development of effective personalised therapies for various retinal degenerative diseases, which can restore vision and improve the quality of life to an extent previously unknown to patients with these conditions. However, research and revision are needed to turn these tactics into safe and successful healthcare treatments.

Safety and efficacy considerations for retinal regeneration using pluripotent stem cells

The treatment of retinal degenerative illnesses may be greatly improved by using pluripotent stem cells, such as iPSCs or ESCs, for retinal regeneration therapy. However, before utilising this strategy in clinical practice, safety and efficacy concerns must be resolved [30].

Tumorigenic Potential

The possibility of tumorigenicity is one of the safety issues with pluripotent stem cell treatment. All three germ layers' worth of cells can combine to form teratomas when stem cells possess pluripotency. Strict quality control methods and in-depth characterization of retinal cells produced from pluripotent stem cells are necessary to lower this danger [31].

Marker-based methods like flow cytometry and immunohistochemistry are used to identify pluripotent markers (e.g., OCT4, NANOG, and SOX2) and the absence of undifferentiated pluripotent stem cells in the final cell population. Besides, genomic and epigenomic analyses can reveal any genomic abnormalities or reactivation of pluripotency-associated genes that would enhance tumorigenicity [32].

Differentiation treatments, such as embryoid body culture, which results in restricted lineage commitment, and fluorescence-activated cell sorting (FACS)-based cell purification protocols decrease the risk of cancer development.

Immunological Rejection

One limitation of cell-based therapies is that the recipient's immune system may not accept transplanted cells generated from pluripotent stem cells because the recipient could perceive them as foreign bodies and reject them. Allogeneic and xenogeneic sources lead to immunogenicity within two categories of cell sources.

This can be done by employing strategies like creating autologous iPSCs, which lowers the chances of immune recognition and rejection when implanted into a patient [33].

However, because it takes time and money to create patient-specific iPSCs, autologous transplantation could only be an option for a select few individuals. Although long-term immunosuppression may have negative effects, immunosuppressive medications can also reduce the immunological response in the receiver. Therefore, there is significant research being undertaken to create techniques to improve immunological tolerance and decrease immunogenicities, such as genetic engineering methods to change the cell surface antigens or use immunomodulatory substances [34].

Long-Term Integration and Functionality

Transplanted cells must integrate into the host retina, create functional connections, and sustain long-term functioning for retinal regeneration therapies to be effective. The stage of retinal development at the time of transplantation, the maturity of the transplanted cells, and the host retina's microenvironment are a few factors that affect how well-transplanted cells integrate and function [35].

Transplanted cells may be evaluated for integration by utilising molecular indicators of functional integration and synaptic connection. Such synaptic proteins such as PSD95 and synaptophysin may be found by immunohistochemical investigation, showing synaptic connections with host neurons. Electrophysiological methods can be used to monitor the electrical activity and responsiveness of transplanted cells, such as patch-clamp recordings or electroretinography (ERG) [36]. The restoration of visual function can also be evaluated using visual behavioural tests in animal models.

It may be necessary to optimise the culture conditions during cell differentiation to produce more mature cell types to promote long-term integration and functioning. As mentioned, encapsulation strategies or scaffold-based methods can give transplanted cells the physical support and signals they need to integrate and mature inside the host retina.

Clinical and Pre-clinical Studies

Before putting pluripotent stem cell-based retinal regeneration treatments into clinical use, thorough pre-clinical research and clinical trials are required to assess safety and effectiveness.

Animal models of retinal degeneration are frequently used in pre-clinical investigations to evaluate the viability, integration, and functioning of transplanted cells. Numerous methods are used, including electrophysiological, immunohistochemistry, genetic analysis, and visual behavioural testing, as was previously mentioned. Animal models help improve transplantation techniques and provide a better understanding of the therapy's possible advantages and hazards [37]. Clinical studies are carried out to assess the therapy's effectiveness and safety in human patients. While Phase II and III studies evaluate effectiveness and long-term effects, Phase I trials concentrate on safety and dose calculation. In order to evaluate the treatment potential and address any safety issues, these studies must carefully identify the patients, monitor their visual function, and provide long-term follow-up [38].

Pluripotent stem cell approaches for retinal regeneration have significant potential for treating retinal degenerative disorders. However, safety and effectiveness issues are of utmost significance. Important considerations for effectively applying these treatments include their tumorigenic potential, immunological rejection, long-term integration and functioning, and thorough clinical and pre-clinical research. Pluripotent stem cell-based retinal regeneration treatments must be carefully characterised, purified, immunomodulated, and optimised, and safe as well as approved efficacious transplantation methods must be used to minimise hazards and guarantee safety and effectiveness.

Future directions and challenges in retinal regeneration therapy

Pluripotent stem cell-based retinal regeneration treatment has enormous potential for treating retinal degenerative disorders. Several upcoming areas and problems must be addressed as the discipline develops to maximise treatment benefits.

Optimisation of Differentiation Protocols

Differentiation methods must be optimised for pluripotent stem cells to differentiate between mature and functioning retinal cell types. Current procedures frequently produce various cell types at various stages of maturity. For retinal regeneration treatment to be effective, these methods must be improved to produce a certain cell type with a set of traits.

Different approaches may be used to improve differentiation procedures. These include enhancing the formation of desirable retinal cell types by modifying the signalling networks involved in retinal development, such as the Wnt, Notch, and Hedgehog pathways. Additionally, retinoic acid, brain-derived neurotrophic factor (BDNF), and basic fibroblast growth factor (bFGF) can all help to promote the maturation and differentiation of particular retinal cell types [39].

Incorporating 3D culture methods or organoid models can also help pluripotent stem cells differentiate into organised retinal tissue structures by better simulating the intricate retinal microenvironment. This strategy can enable the production of particular retinal cell types with enhanced functioning and offer a more realistic picture of in-vivo retinal development.

Enhanced Functional Integration

Transplanted cells must successfully integrate into the host retina and form adequate connections with the pre-existing retinal circuitry to restore vision properly. A significant obstacle in the treatment of retinal regeneration is improving the functional integration of transplanted cells.

Functional integration depends on enhancing synaptic connections between transplanted cells and host neurons. Synaptic development may be facilitated, and the functional connection between transplanted cells and host retinal neurons can be strengthened using techniques including boosting synaptic protein expression, timing the transplantation perfectly, and supplying the right environmental signals.

In order to promote the integration and functioning of transplanted cells and provide them with the ability to respond to visual cues, optogenetic procedures that insert light-sensitive proteins into transplanted cells or the development of electrical stimulation techniques can be used [40].

Regulatory Considerations

Regulatory issues are crucial in guaranteeing the safety and effectiveness of retinal regeneration therapy as it develops and finds use in clinical settings. To approve novel medications, regulatory agencies like the FDA in the United States want solid pre-clinical and clinical data.

Manufacturing procedures must follow good manufacturing practices (GMP) regulations to ensure dependable and secure cell product manufacture. Stringent characterisation and safety tests must be implemented to evaluate the identity, purity, and safety of the retinal cells produced from pluripotent stem cells. Long-term safety monitoring and post-marketing surveillance are essential to assess the possible dangers of retinal regeneration treatment. Close cooperation between researchers, doctors, and regulatory bodies is crucial to successfully traverse the regulatory landscape and ensure the effective translation of new medicines [41].

Combination Therapy and Complementary Approaches

The effectiveness of retinal regeneration therapy can be increased by combining treatments and other strategies. Addressing various areas of retinal degeneration and encouraging synergistic effects entail combining a number of therapy methods. Combination treatment may entail the sequential administration of various medications or the co-transplantation of various cell types. For instance, the survival and integration of transplanted cells can be improved by combining retinal cell transplantation with neuroprotective techniques or anti-inflammatory drugs [42].

Combining several treatment modalities, such as optogenetic stimulation with cell transplantation or gene therapy that targets certain genetic mutations with cell-based therapies, can give additional advantages and enhance overall therapeutic results. Additionally, combining retinal regeneration therapy with the promise of cutting-edge technologies like nanotechnology, gene editing, and tissue engineering can improve cell survival, fusion, and functioning even more [43].

Pluripotent stem cell-based retinal regeneration treatment has a bright future, but several issues must be resolved to maximise therapeutic results. The main emphasis areas include enhancing the functional integration of transplanted cells, regulatory concerns, and the investigation of combination treatment and complementary techniques. Optimising differentiation protocols to produce mature and functional retinal cell types is also important. For retinal regeneration treatment to advance clinical applications, restore eyesight, and enhance the quality of life for people with retinal degenerative illnesses, it must be developed via ongoing research, cooperation, and new tactics.

Findings from multiple studies are listed in Table 2.

**Table 2:** Findings from various sources in tabulated format along with the year of publication RPE: retinal pigmentary epithelium; PSCs: pluripotent stem cells; AMD: age-related macular degeneration; STGD1: Stargardt type 1 disease; RP: retinitis pigmentosa; PR: photoreceptor; BM: basement membrane

Author	Year	Summary/findings
Napoli et al. [1]	2018	Gave an insight into the intrinsic power of stem cells and their capability to divide and act as a regenerative catalyst for organogenesis rather than replacing the organ.
Jin et al. [2]	2019	This work sheds light on and explains why non-neuronal retinal pigment epithelial cells produced from pluripotent stem cells were the first to be effectively used in human clinical trials.
Website [3]	2023	This article provided us with insight into childhood blindness and how to detect it.
Peichl [4]	1989	This gives an insight into the structural organisation of the retina and its functions.
Wert et al. [5]	2014	This article gives us a brief insight into the mechanism and pathophysiology of retinal degeneration.
Scuteri et al. [6]	2019	This cohort study gave an insight into the effects of diabetic retinopathy on the development of retinal degeneration.
Jin et al. [7]	2021	This study provides an overview of the difficulties associated with employing induced pluripotent stem cells for retinal regeneration.
Ahmed et al. [8]	2021	This work demonstrates that photoreceptor cell transplantation and RPE produced from PSCs provide promising new avenues for treating retinal disorders, including AMD, STGD1, and RP, among others.
Idelson et al. [9]	2009	This study establishes the possibility of using human embryonic stem cells (hESCs) as an endless supply of RPE cells for transplanting under blinded circumstances.
Romito and Cobellis [10]	2016	This demonstrates that the future for treating and maybe curing human illnesses lies in stem cell research. PSCs are particularly interesting in situations where it is challenging to access, grow, or drive functioning adult stem cell types because of their ability to differentiate into a broad variety of cell types.
Zheng [11]	2016	This paper gives an insight into the ethical concerns that are associated with using stem cells for the regeneration of organs or creating organoids.
Ohtsuka and Dalton [12]	2008	This work provides us with information on the maturation of human embryonic stem cells and their potential applications in the future.
Weed and Mills [13]	2017	This research sheds light on the difficulties associated with employing stem cells to promote organ regeneration.
Gonzalez-Cordero et al. [14]	2013	This work unequivocally demonstrates that photoreceptors for retinal cell transplantation may be obtained from embryonic stem cells.
Yuan et al. [15]	2022	Our research tools for treating retinal illnesses are retinal organoids. They enable us to investigate the viability of gene therapy, replicate disease pathophysiology and phenotypes in vitro, and get a deeper understanding of the formation and maturation of the retina.
Deochand et al. [16]	2016	According to this article, planarian eye regeneration's temporal regulation is strictly regulated and immune to changes in the kind of damage.
Stenkamp [17]	2015	This study summarised the principles of retinal neurogenesis and the process of organogenesis and described some of the important molecular components that are essential for retinal development in order to offer an overview of retinal development.
Oh and Jang [18]	2019	This work demonstrates that the main obstacles to the therapeutic use of PSC-derived cells are straightforward, scalable differentiation processes with high purity; transcription factor-directed PSC differentiation techniques represent a viable approach for next-generation cell therapy.
Gonzalez-Cordero et al. [19]	2017	The results of this study suggest the possibility of using photoreceptor transplantation to treat retinal degeneration by showing that pure human long/medium cones can survive and integrate into the adult mouse retina.
Seritrakul and Gross [20]	2019	This work provides an overview of current research on the genetic and epigenetic control of zebrafish retinal development.
Raeisossadati et al. [21]	2021	Understanding how epigenetic pathways support regenerative responses and if they may be induced in non-regenerative mammals is provided by this work, which could be helpful in the search for innovative treatments to treat retinal diseases.
Maeda et al. [22]	2022	This paper gives a summary of practically using stem cell-based therapies for retinal degeneration.
Scruggs et al. [23]	2019	The results of this work offer a wide range of applications in therapies that depend on subretinal injections, including gene therapy and retinal cell transplantation produced from bolus stem cells.
Khalili et al. [24]	2023	The combination of artificial intelligence (AI) with three-dimensional (3D) bioprinting for the creation of 3D cell scaffolds is suggested in this research as a way to revolutionise the field of retinal tissue engineering and provide new avenues for the development of innovative drug delivery systems for the treatment of visual disorders.
Kador and Goldberg [25]	2012	The benefits of tissue engineering advancements for retinal regeneration are discussed in this research.
Seiler and Aramant [26]	2012	According to this article, retinal progenitor sheet transplantation offers a great model to address concerns regarding the restoration and maintenance of a degenerating retina's functionality.
Cehajic-Kapetanovic et al. [27]	2023	This study provides an overview of the use of electrical devices in tissue rebuilding and healing.
Nair et al. [28]	2021	According to this article, the idea of creating combinations of RPE/PR/BM microscale niches by 3D bioprinting may be a useful strategy to improve visual function by bringing about functional (synaptic) integration with the host neural circuitry.
Dhurandhar et al. [29]	2021	In order to support retinal regeneration therapy, this research offers a genetic screening procedure.
Sharma and Jaganathan [30]	2021	According to this research, MSCs and their byproducts may make better retinal degeneration therapeutic options.
Peterson et al. [31]	2016	According to this study, hPSCs intended for use in cell therapy should be cultivated in a way that reduces the selection of cells with altered genomes.
Kanemura et al. [32]	2014	This work gave us a less tumorigenic approach to cultivating fresh RPE-derived cells.
Pearl et al. [33]	2012	We now have more knowledge on the carcinogenic potential of stem cells and their byproducts thanks to this article.
Liu et al.[34]	2017	In this article, newly produced cells are transplanted into mice, and their reaction to them is studied and simulated by monitoring for rejection.
Ye et al. [35]	2013	The issues surrounding the use of induced pluripotent stem cells for medicinal reasons are covered in this study.
Jarrige et al. [36]	2021	This study discusses and provides information on the potential side effects of stem cell culture, such as the development of teratomas.
Assawachananont et al. [37]	2014	An idea for treating advanced retinal degenerative illnesses by retinal sheet transplantation treatment is presented in this research.
Hirami et al. [38]	2009	According to this research, people may get transplants of a mouse's developing retinal cells to replace a damaged retina.
Di Foggia et al. [39]	2016	This research sheds light on the use of autologous cells for eye transplantation into the same person.
Mandai [40]	2023	The viability of utilising retinal cells to produce organoids is stated in this review.
Jha et al. [41]	2021	This work provides us with a suitable foundation for the safe and effective use of autologous cells in regeneration.
Pennington and Clegg [42]	2016	According to this study, the treatment of AMD and other retinal illnesses may benefit greatly from the use of stem cell-derived RPE monolayers on scaffolds.
Sahle et al. [43]	2019	This study provides an overview of the use of nanotechnology in tissue regeneration.

## Conclusions

Regenerative retinal therapies using pluripotent stem cells offer promising potential for treating retinal degenerative diseases. This research paper has highlighted major results and implications for clinical practice through an extensive literature evaluation, outlining prospects in the area. The review has shed light on the retina's composition and different cell types, the causes of retinal deterioration, and the justification for using pluripotent stem cells in regenerative treatments. Differentiation techniques have been investigated to improve the production of particular retinal cell types, including genetic modification and epigenetic regulation. Techniques for enhancing retinal integration and functioning, including subretinal injections, encapsulation, scaffold-based methods, and cell sheet transplantation, are effective. Strategies used in bioengineering, such as biomaterials and growth factors, can improve the milieu in which transplanted cells will thrive. Safety aspects have been carefully addressed to emphasise the significance of meticulous characterisation and immunomodulatory techniques, including tumorigenic potential, immunological rejection, and long-term integration and functioning. The effects on clinical practice are substantial.

For people with retinal degenerative illnesses, retinal regeneration treatments can restore vision and enhance quality of life. Before these treatments can be extensively used, however, regulatory issues and obstacles associated with enhancing functional integration and improving differentiation techniques must be resolved. Future perspectives in the field include enhancing functional integration of transplanted cells, addressing regulatory issues, and investigating combination therapy and complementary approaches. Future perspectives include improving differentiation protocols to produce mature and functional retinal cells. In conclusion, this study review article emphasises the potential developments in pluripotent stem cell-based regenerative retinal therapy. The results highlight the opportunity to restore eyesight and enhance patient outcomes. Regenerative retinal treatments have the potential to alter clinical practice and offer hope to those suffering from retinal degenerative disorders by tackling the hurdles and embracing future research initiatives.
